# Data Quality Monitoring for the Hadron Calorimeters Using Transfer Learning for Anomaly Detection

**DOI:** 10.3390/s25113475

**Published:** 2025-05-31

**Authors:** Mulugeta Weldezgina Asres, Christian Walter Omlin, Long Wang, David Yu, Pavel Parygin, Jay Dittmann, the CMS-HCAL Collaboration

**Affiliations:** 1 Centre for Artificial Intelligence Research, Department of Information and Communication Technology, University of Agder, 4879 Grimstad, Norway; 2 Department of Physics, University of Maryland, College Park, MD 20742, USA; long.wang@cern.ch; 3 Department of Physics, Brown University, Providence, RI 02912, USA; david_yu@brown.edu; 4 Department of Physics and Astronomy, University of Rochester, Rochester, NY 14627, USA; pavel.parygin@cern.ch; 5 Department of Physics, Baylor University, Waco, TX 76706, USA; jay_dittmann@baylor.edu

**Keywords:** transfer learning, anomaly detection, spatio-temporal, deep learning, autoencoder, high-dimensional data, data quality monitoring, Compact Muon Solenoid, LHC

## Abstract

The proliferation of sensors brings an immense volume of spatio-temporal (ST) data in many domains, including monitoring, diagnostics, and prognostics applications. Data curation is a time-consuming process for a large volume of data, making it challenging and expensive to deploy data analytics platforms in new environments. Transfer learning (TL) mechanisms promise to mitigate data sparsity and model complexity by utilizing pre-trained models for a new task. Despite the triumph of TL in fields like computer vision and natural language processing, efforts on complex ST models for anomaly detection (AD) applications are limited. In this study, we present the potential of TL within the context of high-dimensional ST AD with a hybrid autoencoder architecture, incorporating convolutional, graph, and recurrent neural networks. Motivated by the need for improved model accuracy and robustness, particularly in scenarios with limited training data on systems with thousands of sensors, this research investigates the transferability of models trained on different sections of the Hadron Calorimeter of the Compact Muon Solenoid experiment at CERN. The key contributions of the study include exploring TL’s potential and limitations within the context of encoder and decoder networks, revealing insights into model initialization and training configurations that enhance performance while substantially reducing trainable parameters and mitigating data contamination effects.

## 1. Introduction

Spatio-temporal (ST) anomaly detection (AD) a promising monitoring application of deep learning (DL) in several fields [1,2,3,4,5,6]. A unique quality of ST data is the presence of dependencies among measurements induced by the spatial and temporal attributes, where data correlations are more complex to capture using conventional techniques [1]. A spatio-temporal anomaly can thus be defined as a data point or cluster of data points that violate the nominal ST correlation structure of the normal data points. DL models dominate the recent AD studies, as AD models capture complex structures, extract end-to-end automatic features, and scale for large-volume data sets [5,6,7,8,9,10,11]. AD models can broadly be categorized as: (1) supervised methods requiring labeled anomaly observations [10,11] and (2) unsupervised approaches using unlabeled data, which are more pragmatic in many real-world applications, as data labeling is tedious and expensive [5,6,7,8,9]. Unsupervised AD models trained with only healthy observations are often called semi-supervised approaches [6]. Semi-supervised AD models have accomplished promising performance in reliability, safety, and health monitoring applications in several domains [6,7,8,9].

The deployment of ST DL models in a new environment is often circumscribed by the limited amount of clean data [12]. Data curation for DL modeling remains cumbersome and particularly challenging for temporal data despite abundant availability. Transfer learning (TL) mechanisms have been proposed for DL models to mitigate the challenge of data insufficiency; it accelerates model training and enhances accuracy [10,11,12,13,14,15,16,17,18,19,20,21,22,23]. It aims to achieve in-domain and cross-domain learning by extracting useful information from the model or data of the source task and transferring it to the target tasks [13,20,21,22,23]. TL is widely employed in computer vision (e.g., a large image classifier trained on over 1000 classes with ImageNet1K [24] is fine-tuned to classify a few types of fruit categories) [20] and natural language processing (e.g., BERT [25], initially trained on a massive and diverse text corpus to learn general language features like syntax and semantics, is fine-tuned with smaller task-specific data sets, specialized in question-answering tasks) [21]. It has also been proposed for temporal sensor data related to machine monitoring [14], electricity load monitoring [15], medical applications [16], dynamic systems [17], and ST data for crowd prediction [12,18,26,27], finance [28], environment monitoring [26], and fault diagnosis [11,19]. TL on ST data for AD application remains limited, and deeper investigation on autoencoder (AE) models, assessing both the encoder and decoder networks, is lacking [11,12,18,19,27,28].

Our study discusses ST AD modeling for the *Compact Muon Solenoid* (CMS) experiment at the *Large Hadron Collider* (LHC) [29,30]. The CMS experiment, one of the two high-luminosity general-purpose detectors at the LHC, consists of a tracker to reconstruct particle paths accurately, two calorimeters—the *electromagnetic* (ECAL) and the *hadronic* (HCAL)—to detect electrons, photons, and hadrons, and a *muon* system [30,31]. The CMS experiment employs the *Data Quality Monitoring* (DQM) system to guarantee high-quality physics data through online monitoring that provides live feedback during data acquisition, as well as offline monitoring that certifies the data quality after offline processing [32]. The online DQM identifies emerging problems using reference distributions and predefined tests to detect known failure modes using summary histograms, such as digi-occupancy maps of the calorimeters [33,34]. A digi-occupancy map contains the histogram record of particle hits of the data-taking channels of the calorimeters at the digitization level. The CMS calorimeters may encounter problems during data taking, such as issues with the front-end particle sensing scintillators, digitization and communication systems, back-end hardware, and algorithms [6,35]. These problems are usually reflected in the digi-occupancy maps. The growing complexity of detectors and the variety of physics experiments make data-driven AD systems essential tools for CMS to automate the detection, identification, and localization of detector anomalies [6,36,37,38]. Recent efforts in DQM at CMS have presented DL for AD applications [6,32,35,38,39,40,41]. The synergy in AD has thus far achieved promising results on spatial 2D histogram maps of the DQM for the ECAL [35,38], the muon detectors [40], and ST 3D maps of the HCAL [6].

Further study of TL for ST AD models—often involving combinations of spatial and temporal learning networks—is essential, considering the achievements of TL in other domains [21]. Recent ST DL models are hybrid and commonly made of combinations of variants of convolutional neural networks (CNNs), recurrent neural networks (RNNs), graph neural networks (GNNs), and transformers for various data mining tasks [12,18,27,42,43]. Our study investigates the potential strengths and limitations of TL on high-dimensional ST semi-supervised AD models. Although there are several ST AD architectures in the literature, most operate with 2D spatial data, such as images [10,12,18,26,27], and the ones that incorporate GNN deal with a limited number of nodes ranges from tens to a few hundreds [3,43]. Hence, we limit our discussion to the GraphSTAD system [6]—an AE model made of a CNN, RNN, and GNN operating on high-dimensional 3D spatial data—to deeply investigate the potential of TL for ST AD in the context of calorimeters for the CMS experiment. The GraphSTAD has been proposed for online DQM to automate monitoring of the thousands of HCAL channels through DL in Ref. [6]. The model captures abnormal events using spatial appearance and temporal context on digi-occupancy maps of the DQM. GraphSTAD employs CNNs to capture the behavior of adjacent channels exposed to regional collision particle hits, GNNs to learn local electrical and environmental characteristics due to a shared back-end circuit of the channels, and RNNs to detect temporal degradation on faulty channels [6]. We have transferred a pre-trained GraphSTAD model on the source *HCAL Endcap* (HE) subsystem into another target subsystem of the *HCAL Barrel* (HB) for the TL experiment. The HE and HB are subdetectors of the HCAL; they are designed to capture hadron particles at different positions of the calorimeter. The subdetectors share similarities but also have differences in design, technology, and configuration, such as detector segmentation [44].

Brute-forcing the knowledge from the source into the target, irrespective of their divergence, and thorough investigation of the several network-building modules would cause certain performance degeneration [20,22]. Hence, we have provided insights on TL using various training modes with different network hierarchies of the AE of the GraphSTAD system. The experiment has demonstrated the potential of TL when applied to the feature extraction encoder and the reconstruction decoder networks with different fine-tuning mechanisms on the target dataset. We have also examined the impact of TL with RNN state preservation within and across sliding time windows on ST reconstruction. TL has achieved promising ST reconstruction and AD while reducing the trainable parameters and providing better robustness against anomaly contamination in the training dataset. Our study demonstrates the efficacy of ST TL in overcoming training data sparsity and model training computation.

The key contributions of this study can be summarized as follows:

- This study explores the potential and limitations of TL in the context of high-dimensional ST models for AD application, at scale with 3000–7000 spatial nodes.
- This study, different from existing TL studies, assesses both encoder and decoder networks of a hybrid AE—evaluated on each main building block with various configurations. Related TL studies primarily focus on the feature extraction encoder or fine-tuning the entire network, as highlighted in this study. We present deeper insights and considerations on previously underexplored angles of TL.
- We demonstrate the robustness of TL, with limited training data sets, in improving model accuracy, reducing training parameters, and better mitigation against vulnerability to training data contamination.

We discuss the related work in TL and the CMS DQM system in Section 2. We describe our datasets in Section 3 and the AD and TL methodologies in Section 4. Section 5 presents the performance evaluation and discussion of the results. We provide the conclusion and review the impact of our results in Section 6.

## 2. Background

This section discusses TL in DL models and provides an overview of the DQM system of the CMS experiment.

### 2.1. Transfer Learning on Deep Learning

In the last decade, the effectiveness of DL in handling large datasets has caught the attention of both academia and industry. Its ability to learn nonlinear behavior, along with end-to-end automatic feature extraction, allows it to find complex patterns within high-dimensional large data sets. However, most DL models are complex and require extensive data sizes for modeling, which can be expensive and time-consuming to curate, especially in the case of temporal data. Transfer learning approaches, which incorporate pre-trained models into new tasks, are potential solutions for developing DL models when clean data are limited [14,15,16,17,19,45]. TL is a paradigm where knowledge from a source model or data on different domains (e.g., different data sources or datasets) or tasks (e.g., different model applications) is utilized to improve the efficacy of a target model [13,20,22,23,45].

The TL techniques in the literature can broadly be categorized into various taxonomies [13,20,21]. One of the typical categorizations is based on the similarity of the task and domain between the source and target [13,20,21,22]: (1) inductive TL: the source and target tasks are different, but their domains may remain the same; (2) transductive TL: the tasks remain the same, but the domains are different; and (3) unsupervised TL: similar to inductive transferring on different but related tasks with unlabeled datasets. TL can be carried out on (1) model parameters, where all or some parameters are transferred from a pre-trained source model, and (2) data, where all or part of the source domain data instances are utilized to train the target model [13,21,22]. In this study, TL signifies the use of learned network parameters (*weights* and *biases* from a source model pre-trained on adequate datasets) on a target model for a related task on a different dataset, with or without fine-tuning of the parameters [13]. We refer readers to a survey study in refs. [13,21,22] for further discussion and progress on recent deep TL approaches.

The recent successes of generative models on image and text data have ameliorated the adoption of TL methods for several applications [13,20,21]. The notable contribution of TL is significant in transferring feature extraction networks (*encoders*) that are trained on immense datasets with very expensive computation grids [26,27]. Robustly extracted features reduce the model complexity and training cost of the fine-tuned decision networks while enhancing accuracy [14,16,18,21,26,27]. We refer to this TL mechanism as the freeze and fine-tune approach [13]. Although abundant studies are available for images and language text, TL is relatively less explored for temporal data, such as sensor measurement datasets [19]; TS datasets are often not readily available or accessible on the internet, unlike images and text, and the datasets are often multidimensional and so diverse that they require domain-specific knowledge for data curation and preparation. TL on a temporal data has been investigated in various applications [11,14,15,16,17]. The efforts towards adopting TL for ST data are even more limited [12,18,19,26,27,28]. Hijazi et al. [19] proposed a TL approach that integrates CNN and temporal long short-term memory (LSTM) (referred to as ConvLSTM) to efficiently train a new stability prediction model when the power system undergoes topological changes. Wang et al. [18] applied TL for cross-city crowd-flow prediction where feature extraction ConvLSTM of the forecasting model trained on one city is fine-tuned on another city’s dataset. Wang et al. [12] extended TL on ConvLSTM using a deep adaptation mechanism for crowd-flow prediction. The adaptation network matches the embedding representations of the source and target domain distributions to learn the transferable features between the two domains. Guo et al. [28] fine-tuned an autoencoder for a store recommendation system from a model trained on a different city dataset. Sarker et al. [26] and Natha et al. [27] adopted pretrained 3D CNNs for ST feature extraction to improve anomaly detection on video datasets. Yang et al. [11] proposed unsupervised TL utilizing the fault knowledge learned from labeled sensor fault datasets to perform online anomaly monitoring on unmanned aerial vehicle sensor data. Some studies have employed TL to increase training data from multiple sources and address training with diverse data issues, such as catastrophic forgetting, using different regularization techniques [45]. Recent DL models built on hybrids of CNNs [2,6,18,19,26,27,46,47], RNNs [6,18,19,27,45,48,49], and GNNs [3,6,43,49] have gained momentum for TS and ST data in AD and other data mining applications. Thus far, most TL studies have focused on feature extraction encoding networks and predominantly on forecasting tasks [12,18,19,45]. We have studied the transferability of CNNs, GNNs, and RNNs on both the encoder and decoder networks of an autoencoder and qualitatively evaluated the effectiveness of the TL on ST reconstruction and AD tasks.

### 2.2. The Hadron Calorimeter of the CMS Detector

**Figure 1:**
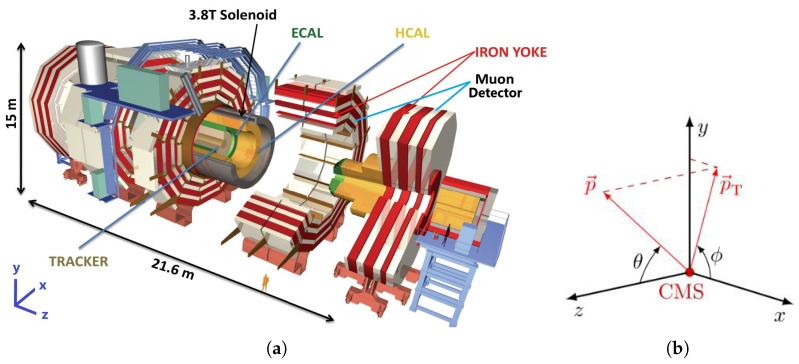
Schematic of the CMS detector: (**a**) CMS with its major systems [50], and (**b**) geometry axes and angles of the CMS with respect to the collision intersection point [51].

Figure 1a shows the CMS experiment and the HCAL detector inside CMS [30,31]. The calorimeters of the CMS detector are highly segmented to improve the accuracy of energy-deposition profile-measurement and particle identification [30,31,50]. The segmentation geometry of the detector is represented using η and ϕ spaces, which correspond to *pseudo-rapidity* and *azimuth*, respectively (as shown in Figure 1b). The *z*-axis lies along the incident beam direction, ϕ is the azimuthal angle between the *x* and *y* axis, and η is calculated from the polar angle $\theta_{cm}$ between the *z* and xy-planes as follows:

(1) $$\eta =-\ln(\tan\left(\theta_{cm}/2\right))$$

where *x*, *y*, and *z* are orthogonal axes of the cylinder, $\theta_{cm}$ is the center-of-mass scattering angle, and ln is a natural log function. The η−ϕ space corresponds to a rectangular coordinate system representing an outgoing particle’s direction from the center of the detector (where the collision occurs). Particles traveling in the same direction lie near each other in η−ϕ space.

**Figure 2:**
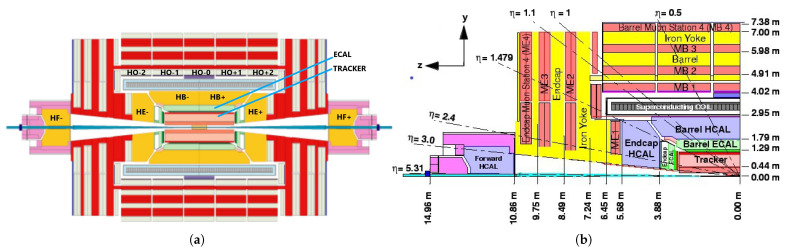
The subdetectors of the HCAL: (**a**) longitudinal view of the HB, HE, HF, and HO subdetectors on CMS [52]; and (**b**) longitudinal view of one quadrant of CMS with segmentation angle specifications of the η, where the origin denotes the interaction point [31,53].

Figure 2a illustrates the four major subdetectors of the HCAL covering different segments in the CMS detector: the HB, the HE, the *HCAL Outer* (HO), and the *HCAL Forward* (HF). Since this study’s datasets are from the LHC Run-2 collision experiment, we will describe the HCAL system configurations from 2018 below. The HB and HE are sampling calorimeters with a brass absorber and active plastic scintillators to measure the energy depositions [31]. The subdetectors surround the ECAL and are fully immersed within the strong magnetic field of the solenoid: the HB are joined hermetically with the barrel extending out to $\left|\eta \right|=1.4$ and the HE covering the overlapping range $1.3<\left|\eta \right|<3.0$ (as shown in Figure 2b). The HF is located 11.2 m from the interaction point and extends the pseudo-rapidity coverage (overlapping with the HE) from $\left|\eta \right|=2.9$ to $\left|\eta \right|=5$. The central shower containment in the region $\left|\eta \right|<1.26$ is improved with the HO, an array of scintillators located outside the magnet.

The front-end electronics of the HCAL, responsible for sensing and digitizing optical signals of the collision particles, are divided into sectors of *readout boxes* (RBXes) that house the electronics and provide voltage, backplane communications, and cooling. Our study’s use cases, the HE and HB, consist of 36 RBXes arranged on the plus (HE[HB]P) and minus (HE[HB]M) hemispheres of the CMS detector. The front-end acquisition systems transmit the photons produced in the plastic scintillators through the wavelength-shifting fibers to the silicon photomultipliers (SiPMs) [HE] or the hybrid photodiode transducers (HPD) [HB] [31]. Each RBX houses frontend electronics that include four digitization readout modules (RMs), the next-generation clock and control module, and the calibration unit [31]. Each RM is made of SiPMs [HE] or HPD [HB], a SiPM control card, and four readout charge integrator and encoder (QIE) cards, each with several QIE chips and field-programmable gate array (FPGA) modules. A QIE chip integrates charge from one SiPM [HE] or HPD [HB] at 40 MHz, and the FPGA serializes and encodes the data from the QIE chips (channels).

### 2.3. CMS Data Quality Monitoring

The collision data of the LHC are organized into runs, where each run contains thousands of luminosity sections (also called lumisections). A lumisection (LS) corresponds to approximately 23 s of data collection and comprises hundreds or thousands of collision events containing particle hit records across the CMS detector. The DQM system in CMS provides feedback on detector performance and data reconstruction; it generates a list of certified runs for physics analyses and stores it in the “Golden JSON” [32]. The DQM employs online and offline monitoring mechanisms: (1) online monitoring is real-time DQM during data acquisition, and (2) offline monitoring provides the final fine-grained data quality analysis for data certification 48 h after the collisions were recorded. The online DQM populates a set of histogram-based maps on a selection of events and provides summary plots with alarms that DQM experts inspect to spot problems. The digi-occupancy map is one of the histogram maps generated by the online DQM, and it contains particle hit histogram records of the particle readout channel sensor of the calorimeters. A digi, also called a hit, is a reconstructed and calibrated collision physics signal of the calorimeter. Several errors can arise in the calorimeter affecting the front-end particle sensing scintillators, the digitization and communication systems, the back-end hardware, or the algorithms. These errors appear in the digi-occupancy map as holes, under- or over-populated bins, or saturated bins. Previous efforts by the authors of refs. [6,32,35,39,40] demonstrate the promising AD efficacy of using digi-occupancy maps for calorimeter channel monitoring using machine learning. Our GraphSTAD has extended the efforts in AD for the HCAL with ST modeling of the 3D digi-occupancy maps of the DQM [6]. The GraphSTAD incorporates both CNNs and GNNs to capture Euclidean and non-Euclidean spatial characteristics, respectively, as well as RNNs for temporal learning for the HCAL channels.

## 3. Dataset Description

**Figure 3:**
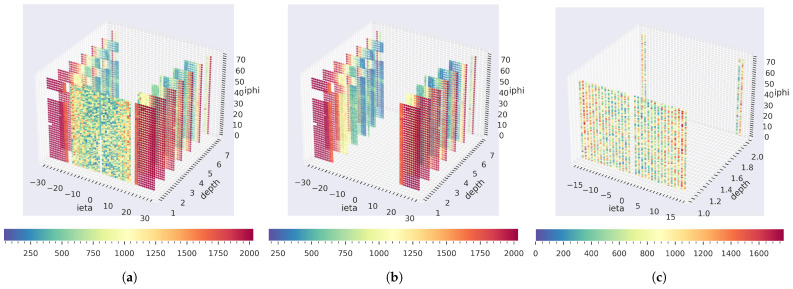
A sample digi-occupancy map (*year = 2018, RunId = 325,170, LS = 15)*: (**a**) digi-occupancy map for the HE and HB together; (**b**) the source system HE channels are placed in $\left|i\eta \right|\in \left[16,\ldots ,29\right]$, iϕ∈[1,…,72], and *depth* ∈[1,…,7]; and (**c**) the target system HB channels are placed in $\left|i\eta \right|\in \left[1,\ldots ,16\right]$, iϕ∈[1,…,72], and *depth* ∈[1,2]. The HE and HB share similarities and differences in tasks, calorimeter technology, and data characteristics. The missing sector at (**b**) corresponds to the two failed HE-RBX sectors during the 2018 collision runs.

We utilized the digi-occupancy data of the online DQM system of the CMS experiment to train and validate our models. The data contain healthy digi-occupancy maps with a 20 fC minimum threshold and were selected from certified good collision runs, as referred to by the “Golden JSON” of CMS. The digi-occupancy datasets were collected in 2018 during the LHC Run-2 collision experiment with a received luminosity per lumisection of up to 0.4 pb^−1^ and up to 2250 events. The source and target datasets contain three-dimensional digi-occupancy maps for the HE and HB subsystems of the HCAL, respectively (as shown in Figure 3).

The digi-occupancy map contains a particle hit count of the calorimeter readout channels for a given period of time. The HCAL covers a considerable volume of CMS and has a fine segmentation along three axes (iη∈[−32,…,32], iϕ∈[1,…,72] and *depth* ∈[1,…,7]). The iη and iϕ denote integer notation of the towers covering ranges of η and ϕ of the CMS detector, respectively [31]. The digi-occupancy measurement corresponds to a hit record of the readout channels at the segmentation positions. The similarities between the source and target datasets and tasks have been established in the literature to be essential factors that impact the performance of TL [54]. The source system HE (as shown in Figure 3b) and the target system HB (as shown in Figure 3c) share a similar task but cover different segments of the HCAL. Another major difference between the HE and HB in the 2018 LHC collision run is the front-end data acquisition optical-to-electrical technology, i.e., the HE was upgraded to SiPMs with QIE11 technology, and the HB utilized HPD with QIE8. We compare the source and target datasets in Table 1.

## 4. Methodology

This section presents the GraphSTAD modeling and the experimental setups for the transfer learning study.

### 4.1. Data Preprocessing

This section describes the data preprocessing stages of the proposed approach, i.e., digi-occupancy renormalization and graph-adjacency matrix generation.

#### 4.1.1. Digi-Occupancy Map Renormalization

**Figure 4:**
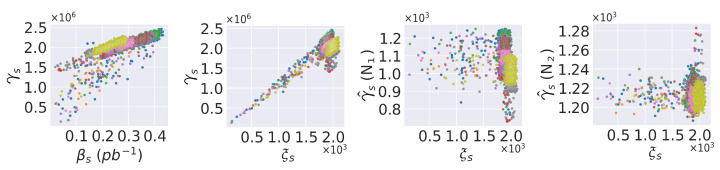
Total digi-occupancy data distribution of the HB and run settings per map (*s*): the received luminosity ($\beta_{s}$) and the number of events ($\xi_{s}$). $N_{1}$ is the renormalization of $\gamma_{s}$ based on $\xi_{s}$, and $N_{2}$ is the reversible renormalization based on the median γ along the iϕ axis. The colors correspond to different collision runs.

We apply digi-occupancy renormalization in the data preprocessing stages to normalize the values for the variation in the luminosity and the number of event configurations in the collision experiments [6]. The digi-occupancy (γ) map data of the HCAL vary with the received luminosity (β) and the number of events (ξ) (as shown in Figure 4). The per-channel $\gamma_{s}\left(i\right)$ can range $\gamma_{s}\left(i\right)=\left[0,\xi_{s}\right]$, where *s* denotes the $s^{\mathrm{th}}$ LS (3D map) in the dataset and *i* denotes the $i^{\mathrm{th}}$ channel in the $s^{\mathrm{th}}$ map. $\xi_{s}$ is usually adjusted with $\beta_{s}$, but not always. β and γ are retrieved from different systems on the existing CMS system; directly accessing β for real-time γ AD monitoring requires further effort. We renormalize the maps ($\gamma_{s}\to {\hat{\gamma }}_{s}$) based on $\xi_{s}$ to obtain consistent interpretation of the γ maps across lumisections:

(2) $${\hat{\gamma }}_{s}=\frac{\gamma_{s}}{\xi_{s}}$$

The renormalization of γ with only ξ does not entirely avoid the data distribution variations across collision runs, and the distribution shifts and unpredictable spikes due to $\beta_{s}$ may affect the AD model training performance on the ST data (see Figure 4). We employ additional reversible renormalization (RN) before and after invoking the AD model to mitigate the non-linearity of the γ ST data. The renormalization exploits the symmetric property of the iϕ axis (the γ channels are less diverse along the iϕ axis); it divides the γ channels per each iη and depth coordinate by the median values along the iϕ axis on the model input and reverses the action on the model output. The remaining impact of $\beta_{s}$ is left to be learned by the AD model from the training data.

#### 4.1.2. Adjacency Matrix Generation

We deployed an undirected graph network G(V,Θ) to represent the HCAL channels in a graph network based on their connections to a shared RBX system. The graph G contains nodes υ∈V, with edges $(\upsilon_{i},\upsilon_{j})\in \Theta$ in a binary adjacency matrix $A\in R^{M\times M}$, where *M* is the number of nodes (the channels). An edge indicates the channels sharing the same RBX as follows:

(3) $$A\left(\upsilon_{i},\upsilon_{j}\right)=\left\{\begin{array}{cc} 1, & \mathrm{if}\;\Omega \left(\upsilon_{i}\right)=\Omega \left(\upsilon_{j}\right)\\ 0, & \mathrm{otherwise} \end{array}\right.$$

where Ω(υ) returns the RBX identification of the channel υ. There are approximately 7000 channels for the HE and 2600 for the HB in a graph representation of the digi-occupancy map. We retrieved the channel-to-RBX mapping from the 2018 HCAL’s calorimeter segmentation map.

### 4.2. Anomaly Detection Mechanism

We denote the AE model of the AD system as F. It takes ST data $X\in R^{T\times N_{i\eta }\times N_{i\phi }\times N_{d}\times N_{f}}$ as a sequence in a time window $t_{x}\in \left[t-T,t\right]$, where $N_{i\eta }\times N_{i\phi }\times N_{d}$ is the spatial dimension corresponding to the iη, iϕ, and depth axes, respectively, and $N_{f}$ is the number of input variables ($N_{f}=1$, as we monitored only a digi-occupancy quantity in the spatial data). The $F_{\theta ,\omega }:X\to \bar{X}$, parametrized by θ and ω, attempts to reconstruct the input ST data X and outputs $\bar{X}$. The encoder network of the model $E_{\theta }:X\to Z$ provides low-dimension latent space, $Z=E_{\theta }\left(X\right)$, and the decoder $D_{\omega }:Z\to \bar{X}$ reconstructs the ST data from Z, $\bar{X}=D_{\omega }\left(Z\right)$ as follows:

(4) $$\bar{X}=F_{\theta ,\omega }\left(X\right)=D_{\omega }\left(E_{\theta }\left(X\right)\right)$$

Anomalies can live for a short time on a single digi-occupancy map, or they can persist over time, affecting a sequence of maps. Aggregated spatial reconstruction error is calculated over a time window *T* using mean absolute error (MAE) to capture a time-persistent anomaly as follows:

(5) $$e_{i,MAE}=\frac{1}{T}\sum_{t^{\prime }=t-T}^{t}\left|x_{i}\left(t^{\prime }\right)-{\bar{x}}_{i}\left(t^{\prime }\right)\right|$$

where $x_{i}\in X$ and ${\bar{x}}_{i}\in \bar{X}$ are the input and the reconstructed digi-occupancy of the ith channel. We standardized $e_{i,MAE}$ to homogenize the reconstruction accuracy variations among the channels when generating the anomaly score $a_{i}$ as follows:

(6) $$a_{i}=\frac{e_{i,MAE}}{\sigma_{i}}$$

where $\sigma_{i}$ is the standard deviation of the $e_{i,MAE}$ on the training dataset. The standardized anomaly score allows us to use a single AD decision threshold α for all the channels in the spatial map. The anomaly flags are generated after applying α to the anomaly scores ($a_{i}>\alpha$). The α value can be tuned to control the detection sensitivity.

**Figure 5:**
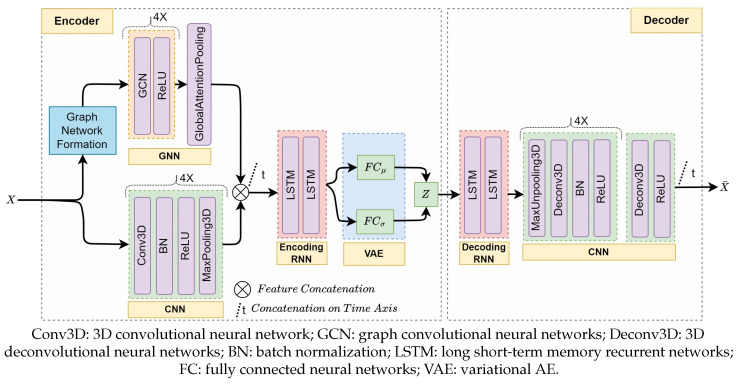
The architecture of the proposed AE for the GraphSTAD system [6]. The GNN and CNN provide spatial feature extraction for each time step, and the RNN network captures the temporal behavior of the extracted features. The feature extraction $E_{\theta }$ incorporates the GNN for back-end physical connectivity among the spatial channels, CNN for regional spatial proximity of the channels, and RNN for temporal behavior extraction. $D_{\omega }$ contains RNNs and deconvolutional neural networks to reconstruct the ST input data from the low-dimensional latent features.

The use-case GraphSTAD AE model is made of CNN, GNN, and RNN networks; it employs a CNN and GNN with a pooling mechanism to extract relevant features from spatial DQM data followed by RNN to capture the temporal characteristics of the extracted features (see Figure 5). It integrates a variational layer [55] at the end of the encoder for regularization of overfitting by enforcing continuous and normally distributed latent representations [6,36]. We refer readers to ref. [6] for further discussion of the mathematical formulation and architecture of the GraphSTAD model.

We trained the AE on healthy digi-occupancy maps (without significant anomaly contamination, see Section 3) of the target HB system. We normalized the spatial data per channel into a [0,1] range to train the model across the variations in calorimeter channels effectively. We utilized a mean squared error (MSE) loss function as follows:

(7) $$L_{MSE}=\frac{1}{M}\sum_{i}{\left(x_{i}-{\bar{x}}_{i}\right)}^{2}$$

where $x_{i}$ and ${\bar{x}}_{i}$ are the input and the reconstructed values of the normalized $\hat{\gamma }$ of the $i^{\mathrm{th}}$ channel, respectively, and *M* is the total number of channels. The variational layer of the AE (denoted as VAE in Figure 5) regularizes the training MSE loss using the *Kullback–Leibler divergence* (KL) distance $D_{KL}$ [55] to achieve the normally distributed latent space as follows:

(8) $$L=\underset{W\in R}{\mathrm{argmin}}\left\{L_{MSE}-\lambda D_{KL}\left[N\left(\mu_{z},\sigma_{z}\right),N\left(0,I\right)\right]+\rho {∥W∥}_{2}^{2}\right\}$$

where N is a normal distribution with zero mean and unit variance, and ${∥.∥}_{2}^{2}$ is a squared *Frobenius norm* of $L_{2}$ *regularization* for the trainable model parameters *W* [56]. λ=0.003 and $\rho =10^{-7}$ are tunable regularization hyperparameters. We employed the *Adam* optimizer [57] for training.

### 4.3. Transfer Learning Approach

Model parameter TL generally consists of four basic steps: (1) selection of a source task with a related modeling problem and an abundance of data where we can exploit the mapping knowledge from the inputs to outputs, (2) development of the source model that performs well in the source task, (3) transfer source model to target model where whole or part of the source model is employed as part of the target model, and (4) fine-tuning the target model on the target dataset if necessary. We present knowledge transfer on GraphSTAD AE models, i.e., an AD model trained on digi-occupancy maps of the source HE subsystem is transferred to the target HB subsystem. Direct transfer of knowledge from the source into the target, irrespective of their divergence and thorough investigation of the model network layers, would limit the efficacy of TL in the target domain [13,20,23].

**Figure 6:**
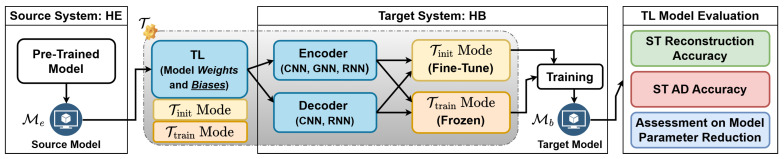
General framework of the proposed transfer learning mechanism.

We have thus investigated several transferring cases when employing TL in two principal model training phases: initialization and training (see Figure 6).

- Init mode ($T_{\mathrm{init}}$): the trainable network parameters (weights and bias) of the source model are transferred into the target model initialization. The target model is further trained on the target HB dataset, resulting in fine-tuning.
- Train mode ($T_{\mathrm{train}}$): The model parameters of the source model are directly reused as the final inference parameters of the target model; the parameters are frozen and excluded from fine-tuning on the target HB dataset.

Let M(Ψ,Ω) be an AD model with parameters Ψ and Ω that represent the model networks that can be affected and not affected by TL, respectively. $M_{e}\left(\Psi_{e},\Omega_{e}\right)$ and $M_{b}\left(\Psi_{b},\Omega_{b}\right)$ are the source and target models for the HE and HB, respectively. The TL modes of T can be formulated mathematically as follows:

(9) $$\begin{array}{cc} T_{\mathrm{init}} & :M_{b}\left(\Psi_{e},\Omega_{b}\right)\underset{\mathrm{fine}-\mathrm{tuning}}{\to }M_{b}\left(\Psi_{e}^{\prime },\Omega_{b}^{\prime }\right)\\ T_{\mathrm{train}} & :M_{b}\left(\Psi_{e},\Omega_{b}\right)\underset{\mathrm{fine}-\mathrm{tuning}}{\to }M_{b}\left(\Psi_{e},\Omega_{b}^{\prime }\right) \end{array}$$

where the superscript ′ denotes the parameters that are updated after fine-tuning the $M_{b}$ model on the target dataset.

$F_{\theta ,\omega }$ of the GraphSTAD is made of CNNs and GNNs with a pooling mechanism to extract relevant features from high-dimensional spatial data, followed by RNNs to capture the temporal characteristics of the extracted features (as shown in Figure 5). Table 2 presents the TL mechanisms that we apply to the different deep networks of the encoder and decoder to study the impacts on ST digi-occupancy map reconstruction and AD accuracy. We also analyze the effects of RNN state preservation within and across time windows. We further investigate variations in training iterations and learning rate scheduling methods. The discussion includes the impact of the TL on reconstruction and AD accuracy, saturation, training stability, and the reduction in the number of model trainable parameters.

The implementation of parameter transferring on DL networks can be accomplished in two ways: (1) start with the source model and then reset (remove and add) the networks that are not included in the TL, and (2) start with the target model with random initialization and update the parameter values of the networks included in the TL from the corresponding source networks. The first approach is widely utilized in DL TL literature and employed for feature extraction; however, it may not be suitable for flexibly choosing layers at different hierarchies, as the target models might have slight variations. Several configuration setups of the AE are derived from the spatial configuration of the input 3D map, which differs for the source HE and target HB systems—e.g., variation in the depth spatial dimension between HE and HB. We have found the second approach more convenient for our study, as we intend to apply TL on different networks of the encoder and decoder of the AE model.

## 5. Results and Discussion

This section will discuss the results of TL on different network layers of the GraphSTAD AE models. We will investigate the effects of TL on reconstruction accuracy, trainable parameter reduction, and AD performance on the target HB digi-occupancy map dataset. We applied TL for model initialization ($T_{\mathrm{init}}$) and training or fine-tuning ($T_{\mathrm{train}}$) on the target HB dataset. We trained the models on NVIDIA Tesla V100 with 4 GPUs using 4000 digi-occupancy maps from LS 1 to 500 and evaluated them on a test set that contains approximately 3000 maps from LS from 500 to 1500. We utilized 20% of the training dataset for validation loss calculation during training to determine the best states for the models. We set the LR at $10^{-3}$ to train the models with five LSs per time window.

### 5.1. Spatio-Temporal Reconstruction Performance

We will discuss below the reconstruction performance (using $L_{MSE}$) of TL applied on spatial (CNNs and GNNs) and temporal (RNNs) learning networks. We will also briefly present comparison results for the LR scheduling choice.

#### 5.1.1. Transfer Learning on Spatial Learning Networks

**Figure 7:**
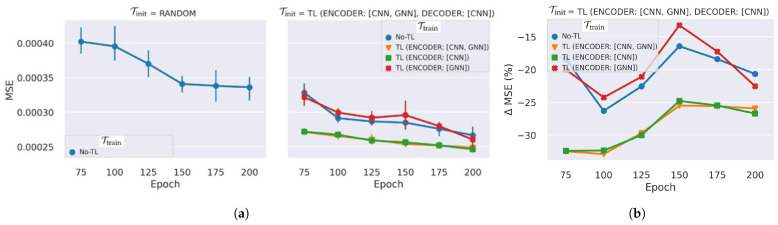
Reconstruction $L_{MSE}$ performance of the TL on spatial networks across different epochs: (**a**) test MSE loss, where the bars show the dispersion of five repeated experiments; (**b**) the average relative difference between the MSE loss and no-TL. TL is applied with $T_{\mathrm{init}}$ on the encoder and decoder using TL-4: ENCODER[CNN, GNN], DECODER[CNN], and $T_{\mathrm{train}}$ on the encoder using TL-1: ENCODER[GNN], TL-2: ENCODER[CNN], and TL-3: ENCODER[CNN, GNN]. The no-TL model starts to saturate at epoch>150.

We have assessed the transferability of the DL AD model at the initialization and inference phases for the spatial learning networks (CNNs and GNNs) on both the encoder and decoder networks on different numbers of training epochs (see Figure 7). The TL has reduced the reconstruction error $L_{MSE}$ of healthy maps by 32.5% to 20.7% when the number of epochs is varied from 75 to 200 (as shown in Figure 7b). The minimum gain of 13% is achieved at epoch 150, just before the performance of the no-TL model starts to saturate. The complete fine-tuning—TL for initialization, followed by fine-tuning the whole network—provided around 20% improvement. The $L_{MSE}$ generally decreases, while the relative TL gain roughly decreases as the epoch increases to 150. The results are not entirely unexpected; the DL models may improve performance as the training epoch increases, reducing the gap caused by the difference in the initialization and training mechanisms. When the epoch increased beyond 150, the randomly initialized model using no-TL achieves only slight improvement, whereas the $L_{MSE}$ continues to drop for the TL models, increasing the relative gain of the TL. The initialization TL on all the spatial learning networks of the AE using $T_{\mathrm{init}}$ = TL-4 and training only the decoder while freezing the encoder using $T_{\mathrm{train}}$ = TL-3 achieves the best improvement, from 26% to 32.5%. The TL gain of the GNNs is limited compared to CNNs; the CNNs are the primary networks that learn the input spatial data, and they have 15 times more parameters than the GNNs in the use-case GraphSTAD AE model. Transferring and freezing the CNNs of the encoder (TL-2 and TL-3) results in stable performance on repeated experiments. Although TL-2 outperforms TL-3 slightly (by 3%) at epoch 200, TL-3 provides computational leverage (7.2%, see Section 5.1.2), bypassing the training overhead of the GNNs.

Table 3 further provides the average and best ST reconstruction performance at epoch=200. Inference TL on the decoder networks without fine-tuning $T_{\mathrm{train}}$ = TL-2_d_ fails to reconstruct the target data adequately. In an AE architecture, the encoder maps the input into low-dimensional latent space (information compression), while the decoder attempts to reconstruct (information expansion) the target data from the latent space. The decoder networks thus require fine-tuning on the target dataset to adjust their parameters to the target reconstruction effectively. Boulle et al. [17] investigated TL and DL for a univariate chaotic time series classification model; they argued that BN without fine-tuning limits the transferability of CNNs. The scaling and shifting parameters for BN and bias parameters are estimated from the training dataset and strongly correlate to the data. We further studied TL on the decoder when the BN layer and the bias parameters of the CNNs are fine-tuned on the target dataset. $L_{MSE}$ is substantially improved by 50% compared to the frozen decoder (see Table 4). However, the error is still 20 times higher than that without TL, indicating that the CNNs of the decoder also require fine-tuning to achieve reasonable accuracy. The results demonstrate the promising leverage of TL for AE model initialization on both feature extraction encoder and reconstruction decoder networks, whereas fine-tuning with the target dataset is essential for the decoder networks.

#### 5.1.2. Transfer Learning on Spatio-Temporal Learning Networks

We investigated TL on the temporal RNNs (LSTM layers) in both the encoder and decoder networks, along with the spatial learning networks (CNNs and GNNs), using $T_{\mathrm{init}}$ = TL-4 and $T_{\mathrm{train}}$ = TL-3—the best performing TL for spatial networks across epochs (see Figure 7).

Table 5 presents $L_{MSE}$ when TL is applied to the ST networks. We evaluated the models by preserving the RNN states across time windows that leverage the accuracy. When the TL involves freezing the RNNs of the decoder ($T_{\mathrm{train}}$ = TL-6), the $L_{MSE}$ improves by 22.6–32.6% while considerably reducing the model trainable parameters by 97.77%, mainly due to the frozen LSTM networks (see Table 5).

**Figure 8:**
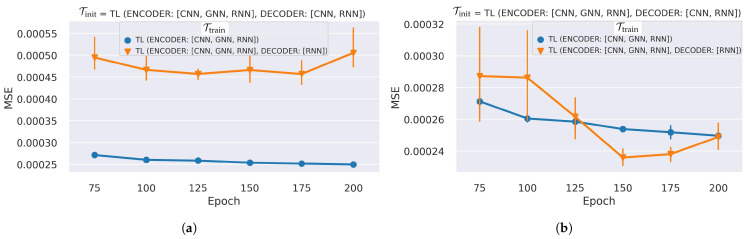
Reconstruction $L_{MSE}$ performance of TL on the ST networks. TL is applied to train the encoder and decoder with TL-5: (ENCODER: [CNN, GNN, RNN]) and TL-6: (ENCODER: [CNN, GNN, RNN], DECODER: [RNN]). The MSE loss in (**a**) non-preserved LSTM states that reset for each time window, and (**b**) preserved LSTM states across consecutive time windows. The bars show the dispersion of five repeated experiments. Lower epochs have higher performance variations among repeated experiments, and the stabilization is better at higher epochs.

**Figure 9:**
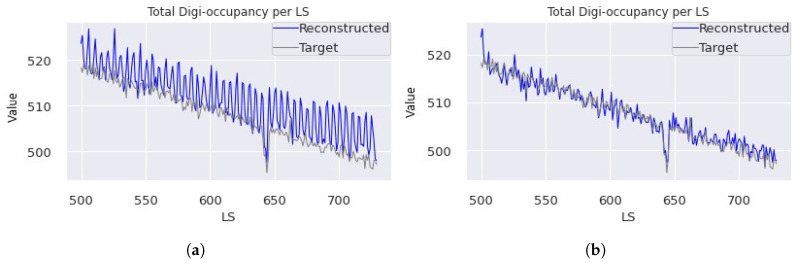
Digi-occupancy map reconstruction on sample ST data from the test set. The model was trained using TL-6, and the inference was executed (**a**) without and (**b**) with LSTM state preservation across time windows. The AE operates on ST γ maps, but the curves in these plots correspond to the aggregate renormalized γ per LS to illustrate the model’s performance in handling the fluctuation across lumisections.

However, the performance of $T_{\mathrm{train}}$ = TL-6 suffers substantially, increasing $L_{MSE}$ by more than 50% if the state memory of the RNNs is not preserved across the sliding time window, i.e., memory reset at every non-overlapping sliding time window start (as shown in Figure 8). Figure 8 presents the $L_{MSE}$ values on multiple epochs when the TL includes the RNNs with and without state preservation across time windows. The plots show a significant enhancement by preserving the states on the frozen decoder RNNs using $T_{\mathrm{train}}$ = TL-6 but a limited impact when the target dataset fine-tunes the decoder RNNs using $T_{\mathrm{train}}$ = TL-5. Figure 9a demonstrates that the TL-6 model struggles to reconstruct the map at the first time step in each sliding time window when states are not preserved across time windows. This is caused by the model’s reliance solely on the input map for the first time-step reconstruction with reset memory states (zeros), while the states are adjusted and improved for the subsequent maps. The reconstruction improves when utilizing previous states, even for the first maps in the time windows (see Figure 9b).

#### 5.1.3. Applying Learning Rate Scheduling

The AE $L_{MSE}$ reaches saturation after epoch>150 when trained without TL (as illustrated in Figure 7a). Learning rate (LR) scheduling mechanisms, e.g., lowering the LR when the loss flattens, or fast convergence methods, could mitigate training stagnation. We have investigated the impact of scheduling on the TL by training the model with super-convergence *one-cyclic* LR scheduling [59]. The LR scheduling sets the LR according to a one-cycle policy that anneals the LR from an initial LR ($init\_lr=4\times 10^{-5}$) to a maximum LR ($max\_lr=10^{-3}$) and then from that maximum LR to a minimum LR ($min\_lr=4\times 10^{-7}$). We utilize a cosine annealing mechanism along with the other settings of the scheduler, such as div_factor=25 and final_div_factor=100, where div_factor determines the initial LR by dividing max_lr, and final_div_factor estimates min_lr by dividing the initial LR. We have kept the default values of the remaining hyperparameters given in the PyTorch = 1.12.0 implementation [59].

Table 6 shows that the LR scheduling has improved the $L_{MSE}$ compared to the fixed LR (provided in Table 5) by 19% for without TL, and 7.1% and 4.3 % for TL with $T_{\mathrm{train}}$ = TL-5, and $T_{\mathrm{train}}$ = TL-6, respectively. The relative progress of the TL is approximately 9% with the LR scheduling, which is lower than the 22.6% achieved with the fixed LR. The results are consistent with Figure 7b, showing a narrowing of the performance difference as the number of epochs increases past epoch>150, with performance saturating for the model without TL, $T_{\mathrm{init}}$ = RANDOM. The cyclic LR scheduling method may require more configuration tuning effort to improve the performance compared to fixed LR or other simpler LR scheduling approaches.

### 5.2. Anomaly Detection Performance

Machine learning studies performed thus far in the CMS DQM system have primarily employed simulated anomaly data to evaluate the efficacy of the developed AD models [6,35]; a small fraction of the DQM data is affected by real anomalies that are inadequate for comprehensive model validation. We validate the AD models on synthetic anomalies simulating real channel anomalies of the HCAL [6]. We have generated synthetic anomalies simulating *dead*, *hot*, and *degraded* channels and injected them into healthy digi-occupancy maps of the test dataset. We formulate the simulated channel anomalies as follows:

(10) $$\gamma_{a}=\left\{\begin{array}{cc} R_{D}\gamma_{h}, & \mathrm{where}\;R_{D}\neq 1\;\mathrm{and}\;\gamma_{a}\leq \xi \\ \gamma_{a}=\xi , & \mathrm{where}\;R_{D}=1\;\mathrm{and}\;\gamma_{h}<\xi \end{array}\right.$$

where $\gamma_{a}\in \left[0,\xi \right]$ and $\gamma_{h}\in \left(0,\xi \right]$ are the digi-occupancy of the generated anomaly channel and its corresponding expected healthy reading, respectively. The $R_{D}$ is the degradation factor, and the channel anomalies are defined as follows:

(11) $$\begin{array}{cc} \mathrm{Dead} & :\gamma_{a}=0,\;\mathrm{using}\;R_{D}=0\\ \mathrm{Degraded} & :0<\gamma_{a}<\gamma_{h},\;\mathrm{using}\;0<R_{D}<1\\ \mathrm{Noisy}\;\mathrm{hot} & :\gamma_{h}<\gamma_{a}\leq \xi ,\;\mathrm{using}\;R_{D}>1\\ \mathrm{Fully}\;\mathrm{hot} & :\gamma_{h}<\gamma_{a}=\xi ,\;\mathrm{using}\;R_{D}=1 \end{array}$$

The algorithm that generates the synthetic anomaly samples involves three steps [6]: (1) selection of a random set of LSs from the test set, (2) random selection of spatial locations φ for each LS, where φ∈[iη×iϕ × *depth*] on the HB axes (see Figure 3c), and (3) injection of the simulated anomalies into digi-occupancy maps of the LSs. The simulated anomalies include dead, degraded, noisy hot, and fully hot channels. For consistency, we have kept the same spatial locations for all the anomaly types. We have evaluated the performance on several classification metrics using three anomaly thresholds set to capture 90%, 95%, and 99% of the injected anomalies.

We evaluate the AD accuracy on 14,000 digi-occupancy maps (2000 maps for each anomaly type) for the dead ($R_{D}=0\%$), decaying anomalies ($R_{D}=\left[80\%,60\%,40\%,20\%\right]$), noisy hot ($R_{D}=200\%$), and fully-hot ($\gamma_{a}=\xi$) channels. We investigate persistent channel anomalies that affect consecutive maps in a time window. We have processed 70,000 digi-occupancy maps (with the generated 1.17% abnormal channels) that include five history maps in the time window for each of the 14,000 maps.

We compare the AD performance of models without TL and the best TL from Table 6. The models are denoted as follows:

(12) $$\begin{array}{cc} M_{N} & :T_{\mathrm{init}}\;=\;\mathrm{RANDOM}\;\mathrm{and}\;T_{\mathrm{train}}\;=\;\mathrm{No}\text{-}\mathrm{TL}\\ M_{T} & :T_{\mathrm{init}}\;=\;\mathrm{TL}\text{-}7\;\mathrm{and}\;T_{\mathrm{train}}\;=\;\mathrm{TL}\text{-}6 \end{array}$$

where $M_{N}$ and $M_{T}$ are the models trained without TL and with TL, respectively.

**Figure 10:**
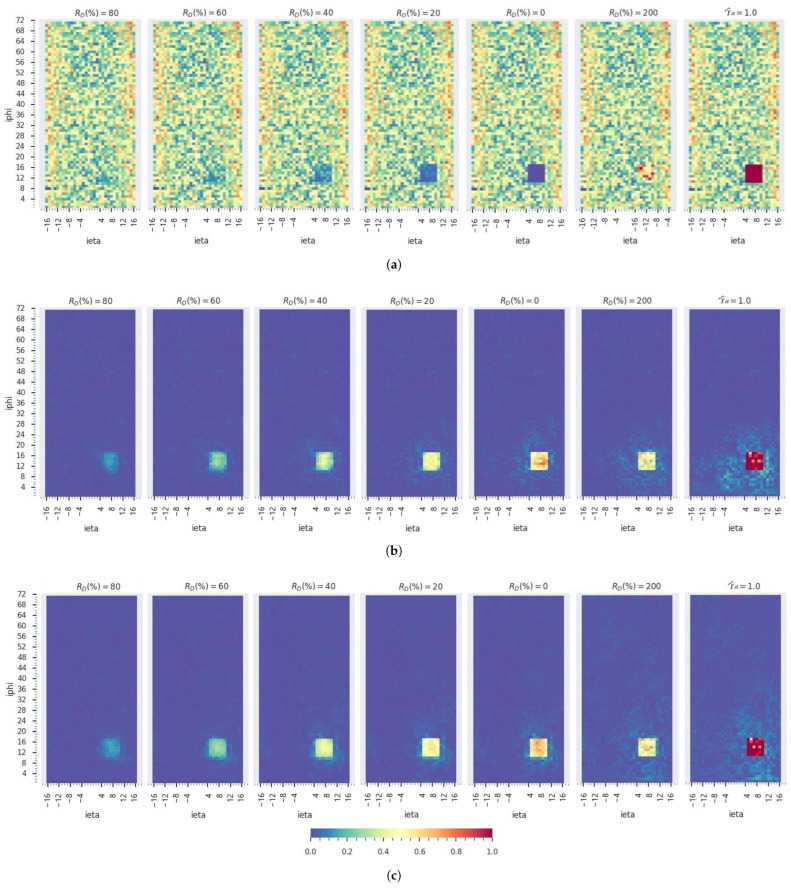
Spatial AD $e_{i,MAE}$ on a sample digi-occupancy map at *depth* =1 with degraded ($0<R_{D}<100\%$), dead ($R_{D}=0\%$), noisy hot ($R_{D}=200\%$), and fully hot (${\hat{\gamma }}_{a}=1.0$) anomaly types: (**a**) renormalized digi-occupancy map with simulated anomaly channels; the reconstruction error maps of the (**b**) $M_{N}$ model and (**c**) the $M_{T}$ model. The anomaly region is localized well with proportional strength to the severity of the anomaly in both models. The $M_{T}$ model has better localization with relatively less dispersion in its anomaly score map.

Table 7 presents the AD accuracy of the models on the dead, degrading, fully hot, and noisy hot channel abnormalities. Both models perform well in the *area under the receiver operating characteristic curve* (AUC) and *false positive rate* (FPR). The TL model significantly improves dead and fully hot channel detection but performs slightly lower for the noisy hot channels. Figure 10 demonstrate the ability of the models to detect and localize the different anomaly types that have been injected at sample channels located at {4<iη<11,11<iϕ<19 *depth*}; the $M_{T}$ model accomplishes better detection on the fully hot channels with less dispersion in its anomaly score values.

**Figure 11:**
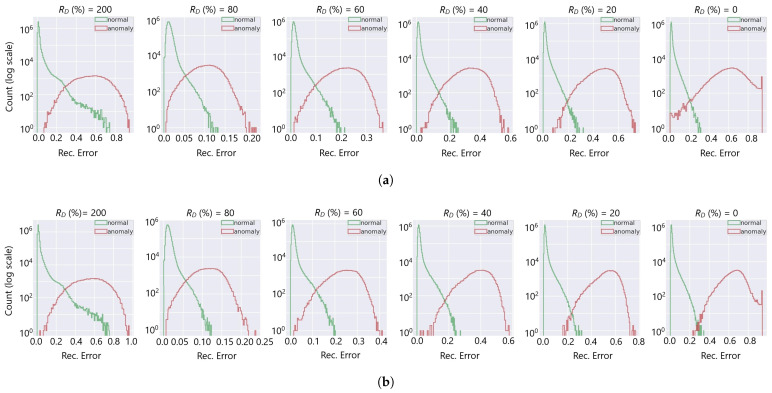
AD reconstruction $e_{i,MAE}$ distribution of healthy and anomalous channels at different degradation rates of the simulated anomalies. The models are (**a**) $M_{N}$ and (**b**) $M_{T}$. The overlap region decreases substantially as the channel deterioration increases for $R_{D}<100\%$. However, the overlap increases for $R_{D}=200\%$, as the error increases for the normal channels due to the correlation to adjacent anomalies (as shown in Figure 12).

**Figure 12:**
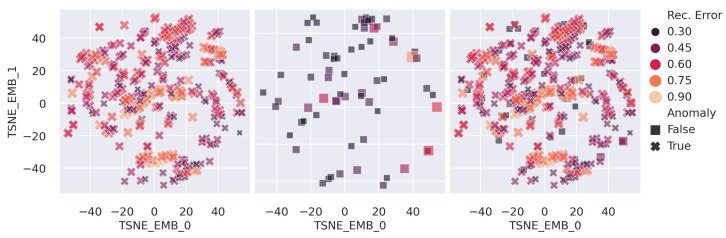
Spatio-temporal location embedding for channels with high $e_{i,MAE}$ in the presence of noisy hot anomalies ($R_{D}=200\%$) with $M_{T}$: (left to right) location embedding for the anomaly channels (Anomaly=True), the normal channels (Anomaly=False), and both, respectively. We applied t-SNE embedding [60] to the channels’ locations (coordinates: LS, iη, iϕ, and *depth*) to generate the 2D representation. The normal channels (Anomaly=False) with high reconstruction error occur near the anomalous channels.

**Figure 13:**
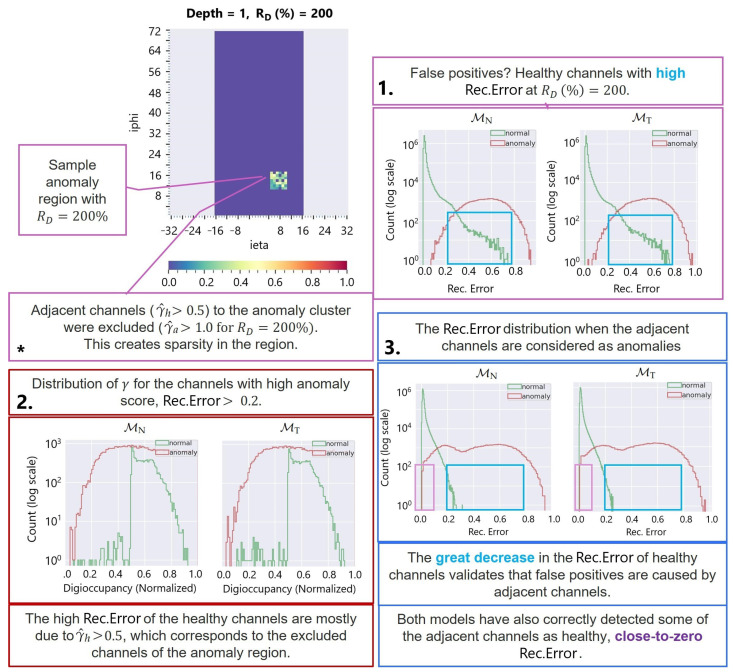
Proximity effect explanation for false positives in noisy hot channel anomaly ($R_{D}=200\%$) detection. The healthy channels with higher AD scores ($e_{i,MAE}>0.2$) belong to the filtered out channels from the anomaly injection $R_{D}\gamma_{h}>\xi$ (see Equation (11)) and generate a high score due to their proximity to the abnormal channels. * Example of anomaly region selection where channels with $R_{D}\gamma_{h}>\xi$ are excluded from the selection to meet the requirement of $\gamma_{a}\in \left[0,\xi \right]$.

Figure 11 portrays the distribution of the AD reconstruction error score ($e_{i,MAE}$) and the overlap region between the healthy and faulty channels at the different degradation rates. We observed an increase in the error score of the healthy channels for the noisy hot channel anomalies at $R_{D}=200\%$. Close investigation reveals that the healthy channels have higher anomaly scores due to their proximity to the abnormal channels (as illustrated in Figure 12); the channels are filtered out from the anomaly simulation generation in Equation (11) due to $\gamma_{a}=R_{D}\gamma_{h}>\xi$. Since channels positioned in proximity to the HB segmentation may share common RBX and are exposed to similar collision particles, the AD AE exploits the correlation for spatial data reconstruction. Figure 13 elaborates further proof of the proximity, showing that the false positive healthy channels with higher anomaly scores ($e_{i,MAE}>0.2$) belong to $R_{D}\gamma_{h}>\xi$.

**Figure 14:**
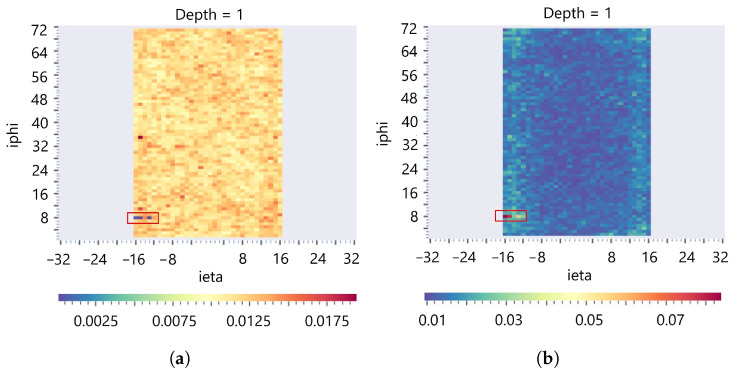
Spatial AD $e_{i,MAE}$ per channel map at *depth* =1 averaged over the training dataset for the (**a**) $M_{N}$ model, and (**b**) $M_{T}$ model. $M_{N}$ reconstructs the real dead channels (in red boxes) as normal, with very low error scores. In contrast, $M_{T}$ produces a high error, which signifies detecting the channels as anomalies.

The degrading and dead channels are another major difference between the $M_{N}$ and $M_{T}$ models. Table 7 shows that the AD slightly deteriorates for the dead channels ($R_{D}=0\%$) compared to the degraded channel at $R_{D}=20\%$, defying the expectation of getting better AD on stronger anomalies. The error score of the $M_{N}$ model drops to zero for dead channels, although the channels have higher error scores at $R_{D}=60\%$ (see Figure 11a); this is influenced by the presence of real dead channels in the training dataset at the location of {iη∈[−16,−15,−13], iϕ=8, *depth* =1} (see Figure 14). Figure 14 depicts that the $M_{N}$ model learned to reconstruct the real dead channels as healthy (very low error score), whereas $M_{T}$ provides a high error, signifying it is detecting the channels as anomalies. The results demonstrate TL robustness when a semi-supervised model’s training dataset is contaminated with real anomalies.

## 6. Conclusions

We have presented transfer learning on anomaly detection models in the context of high-dimensional spatio-temporal autoencoders. We have discussed TL using semi-supervised AD models designed to monitor the Hadron Calorimeter using three-dimensional digi-occupancy maps of the Data Quality Monitoring system. We have successfully transferred the AD model, employing convolutional, graph, and recurrent neural networks, from the source HCAL Endcap to the target HCAL Barrel calorimeter using several ST TL configurations. This study has provided insights into several TL scenarios at the model initialization and training phases in both the encoder and decoder networks. Applying TL to the feature extraction networks of the encoder and inner reconstruction networks of the decoder provided promising results in the ST AD. The approach has also demonstrated potential leverage for small training datasets, a significant reduction in training computation, and an enhancement in robustness against data contamination in the training dataset. In addition to the similarity between the source and target datasets, the choice of model settings, such as the target network layers, the number of training iterations, the learning rate schedule, and the temporal state preservation during inference, can influence the performance of TL. Our study remains relevant to applications in other domains, as it provides essential understanding of TL on widely utilized hybrid neural network layers applied to ST AD datasets. 

## Figures and Tables

**Table 1:** Description of source and target datasets.

Dataset	Sensor Technology	No. of Channels per RBX	No. of RBXes	Calorimeter Segmentation Ranges	Sample Size
Source (HE)	SiPM	192	36	$\left|i\eta \right|\in \left[16,\ldots ,29\right]$, iϕ∈[1,…,72], *depth* ∈[1,…,7]	20,000
Target (HB)	HPD	72	36	$\left|i\eta \right|\in \left[1,\ldots ,16\right]$, iϕ∈[1,…,72], *depth* ∈[1,2]	7000

**Table 2:** Transfer learning experiment configurations.

Config.	Init Mode ($T_{\mathrm{init}}$)		Train Mode ($T_{\mathrm{train}}$)	
	Notation	Description	Notation	Description
1	RANDOM	$M_{b}$ is initialized randomly (weights: using Kaiming uniform [58], and biases: zero)	No-TL	Complete training (fine-tuning)
2	TL-4	$M_{d}$ is initialized randomly, except the spatial learning networks (CNN and GNN) are initialized by TL from $M_{e}$	No-TL	Complete training (fine-tuning)
3			TL-1	GNN of $E_{\theta }$ is frozen (not fine-tuned)
4			TL-2	CNN of $E_{\theta }$ is frozen
5			TL-2_d_	CNN of $D_{\omega }$ is frozen
6			TL-3	CNN and GNN of $E_{\theta }$ are frozen
7	TL-7	All the spatial and temporal learning networks (CNN, GNN, and RNN) of the $M_{b}$ are initialized by TL from $M_{e}$	TL-5	CNN, GNN, and RNN of $E_{\theta }$ are frozen
8			TL-6	CNN, GNN, and RNN of $E_{\theta }$, and RNN of $D_{\omega }$ are frozen

TL: transfer learning is applied. TL-1: ENCODER[GNN], TL-2: ENCODER[CNN], TL-2_d_: DECODER[CNN], TL-3: ENCODER[CNN, GNN], TL-4: ENCODER[CNN, GNN], DECODER[CNN], TL-5: ENCODER[CNN, GNN, RNN], TL-6: ENCODER[CNN, GNN, RNN], DECODER[RNN], TL-7: ENCODER[CNN, GNN, RNN], DECODER[CNN, RNN].

**Table 3:** ST reconstruction $L_{MSE}$ of TL on spatial networks (epoch=200).

$T_{\mathrm{init}}$	$T_{\mathrm{train}}$	$L_{\mathrm{MSE}}↓$	$\Delta L_{\mathrm{MSE}}$ w.r.t $T_{\mathrm{init}}$ = RANDOM↓
Average Score			
RANDOM	No-TL	$3.361\times 10^{-4}$	–
TL-4	No-TL	$2.666\times 10^{-4}$	−20.7%
TL-4	TL-1	$2.604\times 10^{-4}$	−22.5%
TL-4	TL-2	$2.463\times 10^{-4}$	**−26.7%**
TL-4	TL-3	$2.489\times 10^{-4}$	−25.9%
TL-4	TL-2_d_	$1.530\times 10^{-2}$	4452.2%
Best Score			
RANDOM	No-TL	$3.085\times 10^{-4}$	–
TL-4	No-TL	$2.569\times 10^{-4}$	−16.7%
TL-4	TL-1	$2.502\times 10^{-4}$	−18.9%
TL-4	TL-2	$2.420\times 10^{-4}$	**−21.6%**
TL-4	TL-3	$2.451\times 10^{-4}$	−20.5%
TL-4	TL-2_d_	$1.5255\times 10^{-2}$	4844.9%

TL-1: ENCODER[GNN], TL-2: ENCODER[CNN], TL-2_d_: DECODER: [CNN], TL-3: ENCODER[CNN, GNN], TL-4: ENCODER[CNN, GNN], DECODER[CNN]. The **bold font** is the best score and, the down arrow (↓) indicates that lower is better.

**Table 4:** ST reconstruction $L_{MSE}$ of TL for spatial networks ($T_{\mathrm{init}}$ = TL−4, epoch=200, average score).

$T_{\mathrm{init}}$	$T_{\mathrm{train}}$	$L_{\mathrm{MSE}}↓$	$\Delta L_{\mathrm{MSE}}$ w.r.t TL-$2_{d}$↓
TL-4	TL-2_d_	$1.530\times 10^{-2}$	–
TL-4	TL-2_d_/[BN]	$7.200\times 10^{-3}$	−53.0%
TL-4	TL-2_d_/[BN, BIAS]	$7.354\times 10^{-3}$	−51.9%

TL-2_d_: DECODER[CNN], TL-4: ENCODER[CNN, GNN], DECODER[CNN], and / denotes excluding.

**Table 5:** ST reconstruction $L_{MSE}$ of TL on ST networks.

$T_{\mathrm{init}}$	$T_{\mathrm{train}}$	$L_{\mathrm{MSE}}↓$	$\Delta L_{\mathrm{MSE}}$ w.r.t $T_{\mathrm{init}}$ = RANDOM↓	ΔW w.r.t $T_{\mathrm{init}}$ = RANDOM↓
Best Score at epoch=75				
RANDOM	No-TL	$3.826\times 10^{-4}$	–	
TL-4	No-TL	$3.180\times 10^{-4}$	−16.9%	0.00%
TL-4	TL-1	$3.082\times 10^{-4}$	−19.5%	−0.17%
TL-4	TL-2	$2.686\times 10^{-4}$	−29.8%	−2.23%
TL-4	TL-3	$2.705\times 10^{-4}$	-29.3%	−2.39%
TL-7	TL-5	$2.667\times 10^{-4}$	−30.3%	−8.38%
TL-7	TL-6	$2.577\times 10^{-4}$	**−32.6%**	**−97.77%**
Best Score at epoch=200				
RANDOM	No-TL	$3.085\times 10^{-4}$	–	–
TL-4	No-TL	$2.569\times 10^{-4}$	−16.7%	0.00%
TL-4	TL-1	$2.502\times 10^{-4}$	−18.9%	−0.17%
TL-4	TL-2	$2.420\times 10^{-4}$	−21.6%	−2.23%
TL-4	TL-3	$2.451\times 10^{-4}$	−20.5%	−2.39%
TL-7	TL-5	$2.457\times 10^{-4}$	−20.4%	−8.38%
TL-7	TL-6	$2.389\times 10^{-4}$	**−22.6%**	**−97.77%**

TL-1: ENCODER[GNN], TL-2: ENCODER[CNN], TL-3: ENCODER[CNN, GNN], TL-4: ENCODER[CNN, GNN], DECODER[CNN], TL-5: ENCODER[CNN, GNN, RNN], TL-6: ENCODER[CNN, GNN, RNN], DECODER[RNN], TL-7: ENCODER[CNN, GNN, RNN], DECODER[CNN, RNN]. ΔW is the reduction in the number of trainable model parameters. The **bold font** is the best score and, the down arrow (↓) indicates that lower is better.

**Table 6:** ST reconstruction $L_{MSE}$ of TL with LR scheduling mechanism (epoch=200, best score).

$T_{\mathrm{init}}$	$T_{\mathrm{train}}$	$L_{\mathrm{MSE}}↓$	$\Delta L_{\mathrm{MSE}}$ w.r.t $T_{\mathrm{init}}$ = RANDOM↓
RANDOM	No-TL	$2.500\times 10^{-4}$	–
TL-4	No-TL	$2.400\times 10^{-4}$	−4.0%
TL-4	TL-3	$2.460\times 10^{-4}$	−1.6%
TL-7	TL-5	$2.283\times 10^{-4}$	**−8.7%**
TL-7	TL-6	$2.286\times 10^{-4}$	−8.6%

TL-3: ENCODER[CNN, GNN], TL-4: ENCODER[CNN, GNN], DECODER[CNN], TL-5: ENCODER[CNN, GNN, RNN], TL-6: ENCODER[CNN, GNN, RNN], DECODER[RNN], TL-7: ENCODER[CNN, GNN, RNN], DECODER[CNN, RNN]. The **bold font** is the best score and, the down arrow (↓) indicates that lower is better.

**Table 7:** AD performance DQM abnormal channels.

Channel Anomaly Type	FPR (90%) ↓	FPR (95%) ↓	FPR (99%) ↓	AUC ↑
$M_{N}$: $T_{\mathrm{init}}$ = RANDOM and $T_{\mathrm{train}}$ = No-TL				
Degraded ($R_{D}=80\%$)	$6.281\times 10^{-4}$	$1.519\times 10^{-3}$	$8.741\times 10^{-3}$	0.993
Degraded ($R_{D}=60\%$)	$5.991\times 10^{-5}$	$1.438\times 10^{-4}$	$8.242\times 10^{-4}$	**1.000**
Degraded ($R_{D}=40\%$)	$4.881\times 10^{-5}$	$5.628\times 10^{-5}$	$1.466\times 10^{-4}$	**1.000**
Degraded ($R_{D}=20\%$)	$5.870\times 10^{-5}$	$6.273\times 10^{-5}$	$7.342\times 10^{-5}$	**1.000**
Dead ($R_{D}=0\%$)	$6.636\times 10^{-5}$	$7.080\times 10^{-5}$	$8.331\times 10^{-5}$	**1.000**
Noisy hot ($R_{D}=200\%$)	$3.300\times 10^{-4}$	$5.732\times 10^{-4}$	$1.765\times 10^{-3}$	**1.000**
Fully hot ($\gamma_{a}=\xi ,\gamma_{a}>\gamma_{h}$)	$1.220\times 10^{-4}$	$1.317\times 10^{-4}$	$1.606\times 10^{-4}$	**1.000**
$M_{T}$: $T_{\mathrm{init}}$ = TL-7 and $T_{\mathrm{train}}$ = TL-6				
Degraded ($R_{D}=80\%$)	$5.019\times 10^{-4}$	$1.320\times 10^{-3}$	$6.527\times 10^{-3}$	**0.996**
Degraded ($R_{D}=60\%$)	$2.118\times 10^{-5}$	$9.642\times 10^{-5}$	$8.141\times 10^{-4}$	**1.000**
Degraded ($R_{D}=40\%$)	$1.614\times 10^{-6}$	$4.034\times 10^{-6}$	$7.161\times 10^{-5}$	**1.000**
Degraded ($R_{D}=20\%$)	$1.614\times 10^{-6}$	$3.833\times 10^{-6}$	$8.472\times 10^{-6}$	**1.000**
Dead ($R_{D}=0\%$)	$1.815\times 10^{-6}$	$4.236\times 10^{-6}$	$8.472\times 10^{-6}$	**1.000**
Noisy hot ($R_{D}=200\%$)	$1.380\times 10^{-3}$	$2.143\times 10^{-3}$	$4.099\times 10^{-3}$	**1.000**
Fully hot ($\gamma_{a}=\xi ,\gamma_{a}>\gamma_{h}$)	**0.000**	$6.051\times 10^{-7}$	$3.631\times 10^{-5}$	**1.000**

AUC: *area under the receiver operating characteristic curve* and FPR: *false positive rate*. The FPR (ρ%) denotes the FPR score of capturing the ρ% of the anomalies. The **bold font** is the better score between $M_{N}$ and $M_{T}$. The down arrows (↓) indicates that lower is better, and vice versa for th up arrows (↑).

## Data Availability

No new data were created or analyzed in this study. Data sharing is not applicable to this article.

## Supplementary Materials

The following supporting information can be downloaded at: https://www.mdpi.com/article/10.3390/s25113475/s1, Membership of the CMS-HCAL Collaboration.

## Abbreviations

The following abbreviations are used in this manuscript:

AD: Anomaly Detection
AE: Autoencoder
AUC: Area Under the Curve
CMS: Compact Muon Solenoid
CNN: Convolutional Neural Network
DL: Deep Learning
DQM: Data Quality Monitoring
ECAL: Electromagnetic Calorimeter
FC: Fully Connected Neural Network
FPR: False Positive Rate
GNN: Graph Neural Network
GraphSTAD: Graph-based ST AD model
HCAL: Hadron Calorimeter
HB, HE, HF, HO: HCAL Barrel, HCAL Endcap, HCAL Forward, and HCAL Outer subdetectors
HPD: Hybrid Photodiode Transducers
KL: Kullback–Leibler divergence
LHC: Large Hadron Collider
LR: Learning Rate
LS: Luminosity Section
LSTM: Long Short-Term Memory
MAE, MSE: Mean Absolute Error, Mean Squared Error
QIE: Charge Integrating and Encoding
RBX: Readout Box
RNN: Recurrent Neural Network
SiPM: Silicon Photo Multipliers
ST: Spatio-Temporal
TL: Transfer Learning
γ, $\hat{\gamma }$, $\gamma_{h}$, $\gamma_{a}$: Digi-occupancy map, renomalized γ by ξ, healthy γ, anomalous γ
β, ξ: Received luminosity, number of collision events
iη, iϕ, depth: ieta, iphi, and depth axes of the HCAL channels
$F_{\theta ,\omega }$, $E_{\theta }$, $D_{\omega }$: Mathematical notation of an AE model, encoder of $F_{\theta ,\omega }$, decoder of $F_{\theta ,\omega }$
$L_{MSE}$, $e_{i,MAE}$: AE reconstruction MSE loss score, MAE AD score
$R_{D}$: Degradation factor of channel anomaly
$M_{e}$, $M_{b}$: AE model of the TL source HE system, AE model of the TL target HB system
$M_{N}$, $M_{T}$: Model trained without TL, model trained with TL
$T_{\mathrm{init}}$, $T_{\mathrm{train}}$: TL during initialization phase, TL during training phase (frozen parameters)
