# Supplementary material for: Data Quality Monitoring for the Hadron Calorimeters Using Transfer Learning for Anomaly Detection
Source: Sensors (Basel). 2025 May 31;25(11):3475. doi: 10.3390/s25113475 (PMC12158225; doi:10.3390/s25113475)
Supplement: Supplementary file 1 [file sensors-25-03475-s001.zip › sensors-3649535-supplementary.pdf]

THE CMS-HCAL COLLABORATION

A. Gevorgyan[1], A. Petrosyan[1], A. Tumasyan[1], G.A. Alves[2], C. Hensel[2], W.L. Aldá Júnior[3], W. Carvalho[3], J. Chinellato[3,f], C. De Oliveira Martins[3], D. Matos Figueiredo[3], C. Mora Herrera[3], H. Nogima[3], W.L. Prado Da Silva[3], E.J. Tonelli Manganote[3], A. Vilela Pereira[3], M. Finger[4], M. Finger Jr.[4], G. Adamov[5], Z. Tsamalaidze[5,g], K. Borras[6,y], A. Campbell[6], F. Engelke[6,y], D. Krücker[6], I. Martens[6], L. Wiens[6,y], M. Csanád[7], A. Feherkuti[7], S. Lökös[7,v], G. Pásztor[7], O. Surányi[7], G.I. Veres[7], B. Kansal[8], S. Sharma[8], S.B. Beri[9], B. Bhawandeep[9], R. Chawla[9], A. Kalsi[9], A. Kaur[9], M. Kaur[9], G. Walia[9], S. Bhattacharya[10], S. Ghosh[10], S. Nandan[10], A. Purohit[10], M. Sharan[10], S. Banerjee[11], S. Bhattacharya[11], S. Chatterjee[11], P. Das[11], M. Guchait[11], S. Jain[11], S. Kumar[11], M. Maity[11], G. Majumder[11], K. Mazumdar[11], M. Patil[11], T. Sarkar[11] S. Sekmen[12,y], A. Juodagalvis[13], D. Agyel[14], F. Boran[14], S. Damarseckin[14], Z.S. Demiroglu[14], F. Dölek[14], I. Dumanoglu[14,ee], E. Eskut[14], G. Gokbulut[14], Y. Guler[14,ff], E. Gurpinar Guler[14,ff], C. Işik[14], E.E. Kangal[14], O. Kara[14], A. Kayis Topaksu[14], U. Kiminsu[14], G. Onengut[14], K. Ozdemir[14,gg], E. Pinar[14], A. Polatoz[14], A.E. Simsek[14], B. Tali[14,hh], U.G. Tok[14], S. Turkcapar[14], E. Uslan[14], I.S. Zorbakir[14], B. Bilin[15,y], G. Karapinar[15,ii], A. Murat Guler[15], K. Ocalan[15,jj], M. Yalvac[15,kk], M. Zeyrek[15], B. Akgun[16], I.O. Atakisi[16,ll], E. Gülmez[16], M. Kaya[16,ll], O. Kaya[16,mm], S. Tekten[16,nn], E.A. Yetkin[16,dd], T. Yetkin[16,qq], A. Cakir[17], K. Cankocak[17,ee], S. Sen[17,oo], O. Aydilek[18], S. Cerci[18,hh], B. Hacisahinoglu[18], I. Hos[18,pp], B. Isildak[18,qq], B. Kaynak[18], S. Ozkorucuklu[18], O. Potok[18], H. Sert[18], C. Simsek[18], D. Sunar Cerci[18,hh], C. Zorbilmez[18], A. Boyarintsev[19], B. Grynyov[19], L. Levchuk[20], V. Popov[20], P. Sorokin[20], H. Flacher[21], S. Abdullin[22], B. Caraway[22], J. Dittmann[22], K. Hatakeyama[22], A.R. Kanuganti[22], B. McMaster[22], M. Saunders[22], J. Wilson[22], A. Buccilli[23,q], P. Bunin[23,z], S.I. Cooper[23], C. Henderson[23,l], C.U. Perez[23], P. Rumerio[23,t], C. Cosby[24], Z. Demiragli[24], D. Gastler[24], E. Hazen[24], J. Rohlf[24], M. Hadley[25], U. Heintz[25], T. Kwon[25], E. Laird[25], G. Landsberg[25], K.T. Lau[25], X. Yan[25], D. Yu[25,cc], Z. Mao[25], J.W. Gary[26], G. Karapostoli[26,bb], O.R. Long[26], R. Bhandari[27], R. Heller[27], D. Stuart[27], J. Yoo[27,j], Y. Chen[28,n], J. Duarte[28], J.M. Lawhorn[28], M. Spiropulu[28], A. Apresyan[29], A. Apyan[29,c], S. Banerjee[29,d], F. Chlebana[29], Y. Feng[29] J. Freeman[29], D. Green[29], K.H.M. Kwok[29], J. Hirschauer[29], U. Joshi[29], D. Lincoln[29], S. Los[29], C. Madrid[29], N. Pastika[29], K. Pedro[29], W.J. Spalding[29], S. Tkaczyk[29], S. Linn[30], P. Markowitz[30], V. Hagopian[31], T. Kolberg[31], G. Martinez[31], O. Viazlo[31], M. Hohlmann[32], R. Kumar Verma[32], D. Noonan[32], F. Yumiceva[32,e], M. Alhusseini[33], B. Bilki[33], D. Blend[33], K. Dilsiz[33,rr], L. Emediato[33], R.P. Gandrajula[33], M. Herrmann[33], O.K. Köseyan[33], J.-P. Merlo[33], A. Mestvirishvili[33,aa], M. Miller[33], H. Ogul[33,ss], Y. Onel[33], A. Penzo[33], D. Southwick[33], E. Tiras[33,tt], J. Wetzel[33], A. Al-bataineh[34,s], J. Bowen[34,o], C. Le Mahieu[34], J. Marquez[34], W. McBrayer[34], M. Murray[34], M. Nickel[34], S. Popescu[34,r], C. Smith[34], Q. Wang[34], K. Kaadze[35], D. Kim[35], Y. Maravin[35], A. Mohammadi[35,d], J. Natoli[35], D. Roy[35], L.K. Saini[35,f], E. Adams[36], A. Baden[36], O. Baron[36], A. Belloni[36], A. Bethani[36], Y-M Chen[36], S.C. Eno[36], C. Ferraioli[36,i], T. Grassi[36], N.J. Hadley[36], R.G. Kellogg[36], T. Koeth[36], Y. Lai[36], S. Lascio[36], A.C. Mignerey[36], S. Nabili[36], C. Palmer[36], C. Papageorgakis[36], M. Seidel[36,u], L. Wang[36], K. Wong[36], M. D'Alfonso[37], M. Hu[37], B. Crossman[38], J. Hiltbrand[38], M. Krohn[38], J. Mans[38], M. Revering[38], N. Strobbe[38], A. Heering[39], Y. Musienko[39,z], R. Ruchti[39], M. Wayne[39], W. Chung[40], G. Kopp[40], K. Mei[40], C. Tully[40], A. Bodek[41], P. de Barbaro[41], C. Fallon[41], M. Galanti[41], A. Garcia-Bellido[41], A. Khukhunaishvili[41], C-L Tan[41], R. Taus[41], D. Vishnevskiy[41], M. Zielinski[41], B. Chiarito[42], J.P. Chou[42], S.A. Thayil[42], H. Wang[42], N. Akchurin[43], J. Damgov[43], F. De Guio[43,w], S. Kunori[43], K. Lamichhane[43], S.W. Lee[43], T. Mengke[43], S. Muthumuni[43], S. Undleeb[43], I. Volobouev[43], Z. Wang[43], A. Whitbeck[43], G. Cummings[44], S. Goadhouse[44], J. Hakala[44], R. Hirosky[44], D. Winn[45], V. Alexakhin[46], V. Andreev[46], Y. Andreev[46], M. Azarkin[46], A. Belyaev[46], S. Bitioukov[46], E. Boos[46], O. Bychkova[46], M. Chadeeva[46], V. Chekhovsky[46], R. Chistov[46], M. Danilov[46], A. Demianov[46], A. Dermenev[46], M. Dubinin[46,k], L. Dudko[46], D. Elumakhov[46], V. Epshteyn[46], Y. Ershov[46], A. Ershov[46], V. Gavrilov[46], I. Golutvin[46,a†], A. Gribushin[46], A. Kalinin[46,m], A. Kaminskiy[46], A. Karneyeu[46], L. Khein[46], M. Kirakosyan[46], V. Klyukhin[46], O. Kodolova[46,b], V. Krychkine[46], A. Kurenkov[46], A. Litomin[46], N. Lychkovskaya[46], V. Makarenko[46], P. Mandrik[46], P. Moisenz[46,a†], S. Obraztsov[46], A. Oskin[46], P. Parygin[46,x], V. Petrov[46], S. Petrushanko[46], S. Polikarpov[46], E. Popova[46,x], V. Rusinov[46], R. Ryutin[46], V. Savrin[46], D. Selivanova[46], V. Smirnov[46], A. Snigirev[46], A. Sobol[46], A. Stepennov[46,p], E. Tarkovskii[46], A. Terkulov[46], D. Tlisov[46,a†], I. Tlisova[46], R. Tolochek[46], M. Toms[46,h], A. Toropin[46], S. Troshin[46], A. Volkov[46], B. Yuldashev[46], A. Zarubin[46], A. Zhokin[46]

[1]Yerevan Physics Institute, Yerevan, Armenia
[2]Centro Brasileiro de Pesquisas Fisicas, Rio de Janeiro, Brazil
[3]Universidade do Estado do Rio de Janeiro, Rio de Janeiro, Brazil
[4]Charles University, Prague, Czech Republic
[5]Georgian Technical University, Tbilisi, Georgia
[6]Deutsches Elektronen-Synchrotron, Hamburg, Germany
[7]MTA-ELTE Lendület CMS Particle and Nuclear Physics Group, Eötvös Loránd University, Budapest, Hungary
[8]Indian Institute of Science Education and Research (IISER), Pune, India
[9]Panjab University, Chandigarh, India

[10]Saha Institute of Nuclear Physics, HBNI, Kolkata, India
[11]Tata Institute of Fundamental Research-B, Mumbai, India
[12]Kyungpook National University, Daegu, Korea
[13]Vilnius University, Vilnius, Lithuania
[14]Çukurova University, Physics Department, Science and Art Faculty, Adana, Turkey
[15]Middle East Technical University, Physics Department, Ankara, Turkey
[16]Bogazici University, Istanbul, Turkey
[17]Istanbul Technical University, Istanbul, Turkey
[18]Istanbul University, Istanbul, Turkey
[19]Institute for Scintillation Materials of National Academy of Science of Ukraine, Kharkiv, Ukraine
[20]National Science Centre, Kharkiv Institute of Physics and Technology, Kharkiv, Ukraine
[21]University of Bristol, Bristol, United Kingdom
[22]Baylor University, Waco, Texas, USA
[23]The University of Alabama, Tuscaloosa, Alabama, USA
[24]Boston University, Boston, Massachusetts, USA
[25]Brown University, Providence, Rhode Island, USA
[26]University of California, Riverside, Riverside, California, USA
[27]University of California, Santa Barbara - Department of Physics, Santa Barbara, California, USA
[28]California Institute of Technology, Pasadena, California, USA
[29]Fermi National Accelerator Laboratory, Batavia, Illinois, USA
[30]Florida International University, Miami, USA
[31]Florida State University, Tallahassee, Florida, USA
[32]Florida Institute of Technology, Melbourne, Florida, USA
[33]The University of Iowa, Iowa City, Iowa, USA
[34]The University of Kansas, Lawrence, Kansas, USA
[35]Kansas State University, Manhattan, Kansas, USA
[36]University of Maryland, College Park, Maryland, USA
[37]Massachusetts Institute of Technology, Cambridge, Massachusetts, USA
[38]University of Minnesota, Minneapolis, Minnesota, USA
[39]University of Notre Dame, Notre Dame, Indiana, USA
[40]Princeton University, Princeton, New Jersey, USA
[41]University of Rochester, Rochester, New York, USA
[42]Rutgers, The State University of New Jersey, Piscataway, New Jersey, USA
[43]Texas Tech University, Lubbock, Texas, USA
[44]University of Virginia, Charlottesville, Virginia, USA
[45]Fairfield University, Fairfield, USA
[46]Authors affiliated with an institute or an international laboratory covered by a cooperation agreement with CERN.

[a]†Deceased
[b]Also at Yerevan State University, Yerevan, Armenia
[c]Now at Brandeis University, Waltham, USA
[d]Now at University of Wisconsin-Madison, Madison, USA
[e]Now at Northrop Grumman, Linthicum Heights, USA
[f]Now at Gallagher Basset, Schaumburg, USA
[g]Also at Tbilisi State University, Tbilisi, Georgia
[h]Now at Karlsruhe Institute of Technology, Karlsruhe, Germany
[i]Now at Windfall Data, Novato, USA
[j]Now at Korea University, Seoul, Korea
[k]Also at California Institute of Technology, Pasadena, California, USA
[l]Now at University of Cincinnati, Cincinnati, USA
[m]Now at University of Maryland, College Park, Maryland, USA
[n]Now a Massachusetts Institute of Technology, Cambridge, USA
[o]Now at Baker University, Baldwin City, USA
[p]Now at University of Cyprus , Cyprus
[q]Now at Bond, San Francisco, USA
[r]Also at IFIN-HH, Bucharest, Romania
[s]Now at Yarmouk University, Irbid, Jordan

[t]Also at Università di Torino, Torino, Italy
[u]Now at Riga Technical University, Riga, Latvia
[v]Also at Karoly Robert Campus, MATE Institute of Technology, Gyongyos, Hungary
[w]Now at INFN Sezione di Milano-Bicocca, Milano, Italy
[x]Now at University of Rochester, Rochester, New York, USA
[y]Also at CERN, European Organization for Nuclear Research, Geneva, Switzerland
[z]Also at an institute or an international laboratory covered by a cooperation agreement with CERN
[aa]Also at Georgian Technical University, Tbilisi, Georgia
[bb]Now at National Technical University of Athens, Greece
[cc]Now at University of Nebraska, USA
[dd]Also at Istanbul Bilgi University, Istanbul, Turkey
[ee]Also at Near East University, Research Center of Experimental Health Science, Mersin, Turkey
[ff]Also at Konya Technical University, Konya, Turkey
[gg]Also at Izmir Bakircay University, Izmir, Turkey
[hh]Also at Adiyaman University, Adiyaman, Turkey
[ii]Also at Istanbul Gedik University, Istanbul, Turkey
[jj]Also at Necmettin Erbakan University, Konya, Turkey
[kk]Also at Bozok Universitetesi Rektörlügü, Yozgat, Turkey
[ll]Also at Marmara University, Istanbul, Turkey
[mm]Also at Milli Savunma University, Istanbul, Turkey
[nn]Also at Kafkas University, Kars, Turkey
[oo]Also at Hacettepe University, Ankara, Turkey
[pp]Also at Istanbul University - Cerrahpasa, Faculty of Engineering, Istanbul, Turkey
[qq]Also at Yildiz Technical University, Istanbul, Turkey
[rr]Also at Bingol University, Bingol, Turkey
[ss]Also at Sinop University, Sinop, Turkey
[tt]Also at Erciyes University, Kayseri, Turkey
